# Phytocannabinoids: Exploring Pharmacological Profiles and Their Impact on Therapeutical Use

**DOI:** 10.3390/ijms25084204

**Published:** 2024-04-10

**Authors:** Nicoleta Mirela Blebea, Andreea Iulia Pricopie, Robert-Alexandru Vlad, Gabriel Hancu

**Affiliations:** 1 Department of Pharmacology and Pharmacotherapy, Faculty of Pharmacy, “Ovidius” University from Constanța, 900470 Constanța, Romania; nicoleta.blebea@gmail.com; 2 Biochemistry and Chemistry of Environmental Factors Department, Faculty of Pharmacy, “George Emil Palade” University of Medicine, Pharmacy, Science and Technology of Târgu Mureș, 540142 Târgu Mureș, Romania; 3 Pharmaceutical Technology and Cosmetology Department, Faculty of Pharmacy, “George Emil Palade” University of Medicine, Pharmacy, Science and Technology of Târgu Mureș, 540142 Târgu Mureș, Romania; 4 Pharmaceutical and Therapeutic Chemistry Department, Faculty of Pharmacy, “George Emil Palade” University of Medicine, Pharmacy, Science and Technology of Targu Mures, 540142 Târgu Mures, Romania

**Keywords:** phytocannabinoids, cannabidiol, tetrahydrocannabinol, cannabis, cannabinoid receptors

## Abstract

Phytocannabinoids, a diverse group of naturally occurring compounds extracted from the *Cannabis* plant, have attracted interest due to their potential pharmacological effects and medicinal uses. This comprehensive review presents the intricate pharmacological profiles of phytocannabinoids while exploring the diverse impacts these substances have on biological systems. From the more than one hundred cannabinoids which were identified in the *Cannabis* plant so far, cannabidiol (CBD) and tetrahydrocannabinol (THC) are two of the most extensively studied phytocannabinoids. CBD is a non-psychoactive compound, which exhibits potential anti-inflammatory, neuroprotective, and anxiolytic properties, making it a promising candidate for a wide array of medical conditions. THC, known for its psychoactive effects, possesses analgesic and antiemetic properties, contributing to its therapeutic potential. In addition to THC and CBD, a wide range of additional phytocannabinoids have shown intriguing pharmacological effects, including cannabichromene (CBC), cannabigerol (CBG), and cannabinol (CBN). The endocannabinoid system, made up of the enzymes involved in the production and breakdown of endocannabinoids, cannabinoid receptors (CB1 and CB2), and endogenous ligands (endocannabinoids), is essential for preserving homeostasis in several physiological processes. Beyond their effects on the endocannabinoid system, phytocannabinoids are studied for their ability to modify ion channels, neurotransmitter receptors, and anti-oxidative pathways. The complex interaction between phytocannabinoids and biological systems offers hope for novel treatment approaches and lays the groundwork for further developments in the field of cannabinoid-based medicine. This review summarizes the state of the field, points out information gaps, and emphasizes the need for more studies to fully realize the therapeutic potential of phytocannabinoids.

## 1. Introduction

The *Cannabis sativa L.* plant is an annual herbaceous flowering species from the *Cannabaceae* family, originating from Central Asia, which has a rich history of diverse applications throughout the history of humanity. Over centuries, it has been a valuable resource for producing hemp fiber (utilized in clothing, rope, and paper) seeds (consumed as food) and has been served as a medicinal plant. The utilization of *Cannabis* spans a broad spectrum of human activities, from its historical significance in textile and paper production to its modern applications in medicine and recreation [1,2].

Characterized by its high variability in phenotypes, the *Cannabis* plant is classified into three subspecies: *Cannabis sativa* subsp. *sativa*, *Cannabis sativa* subsp. *indica*, and *Cannabis sativa* subsp. *ruderalis* [1,3].

The versatility of the plant and its subspecies has contributed to its enduring relevance across diverse cultures and societies. As global perspectives on *Cannabis* continue to evolve, ongoing research and exploration of its diverse properties are shaping our understanding and expanding the potential applications of this remarkable plant [3,4].

The *Cannabis* plant contains naturally occurring chemical substances called phytocannabinoids. These compounds are unique to the *Cannabis* plant and play a crucial role in the plant’s interactions with the environment, including defense mechanisms against pests and environmental stressors. *Cannabis* plants contain more than one hundred distinct phytocannabinoids, which are known to contribute to the pharmacological effects of the plant. The biosynthesis of phytocannabinoids occurs in glandular trichomes, tiny hair-like structures found on the surface of the *Cannabis* plant. These trichomes produce a resin that contains a rich mixture of cannabinoids, terpenes, and other compounds. The biosynthetic pathway involves enzymes that convert precursor compounds into various phytocannabinoids [5].

**Figure 1:**
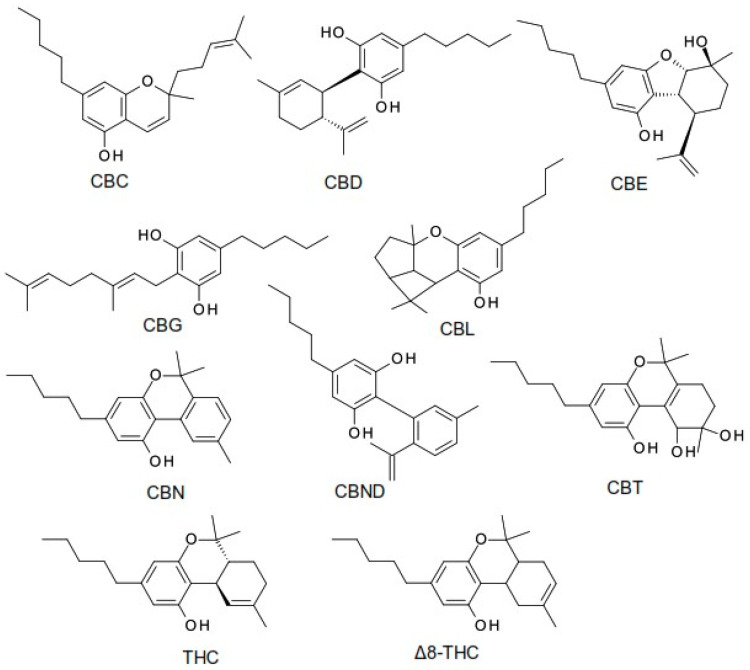
The structure of the most common phytocannabinoids [5].

The term cannabinoid refers to both natural cannabinoids (endocannabinoids, phytocannabinoids) and synthetic cannabinoids that operate on cannabinoid receptors. Phytocannabinoids refer to a group of oxygenated aromatic hydrocarbon metabolites derived from the *Cannabis* plant that contain 21 carbon atoms. Among these, we can identify the two major components in the *Cannabis* plant: the main psychoactive compound, tetrahydrocannabinol (THC), and the non-psychoactive compound, cannabidiol (CBD). Currently, phytocannabinoids are conventionally classified into 11 chemical classes, each named after the “lead” compound: cannabichromene (CBC), cannabidiol (CBD), cannabielsoin (CBE), cannabigerol (CBG), cannabicyclol (CBL), cannabinol (CBN), cannabinodiol (CBND), cannabitriol (CBT), tetrahydrocannabinol (THC), (−)-Δ8-trans-tetrahydrocannabinol (Δ8-THC), and miscellaneous phytocannabinoids (Figure 1) [5,6,7].

The interaction between phytocannabinoids and the endocannabinoid system (ECS) in humans and animals is a key aspect of their pharmacological effects. The ECS is a sophisticated signaling system that is essential for controlling several physiological functions, including mood, hunger, sleep patterns, the immune system, and pain perception. Through their interactions with the ECS’s cannabinoid receptors (CB1 and CB2), phytocannabinoids affect key physiological processes and enhance their potential as therapeutic agents [6,8].

The plant *Cannabis* exhibits an effect called the “entourage effect”, in which the combined action of terpenes and phytocannabinoids results in effects that exceed the sum of their separate contributions. This synergy emphasizes how important it is to consider the entire plant when utilizing cannabis medicinally as opposed to just concentrating on individual cannabinoids [9].

The CB1 and CB2 cannabinoid receptors are recognized as key mediators of the biological effects induced by cannabinoids, whether derived from plants (phytocannabinoids), synthetically produced (synthetic cannabinoids), or naturally occurring in the body (endocannabinoids). Encoded by separate genes on human chromosomes (6q14-q15 for CB1 and 1p36.11 for CB2), these receptors belong to the G protein-coupled receptor family with seven transmembrane domains. They specifically pertain to the Gi/o group (Gi_1_,_2_,_3_; Go_1_,_2_), featuring an extracellular –NH_2_ end and an intracellular –COOH end. Responsible for numerous physiological effects within the ECS, CB1 and CB2 receptors are distributed in both the central nervous system (CNS) and peripheral tissues. Despite sharing 68% similarity in transmembrane domains and 44% overall, the receptors exhibit nuanced pharmacological profiles and expression patterns, allowing for diverse experimental studies, especially in murine models [10,11].

CB1 receptors play a crucial role in transmitting nociceptive information in various tissues and can synergize with opioid receptor agonists. Activating CB1 receptors enhances the release of T-helper 1 cytokines, interferon, interleukin 2, and tumor necrosis factor α, influencing immune and inflammatory processes. CB2 also inhibits adenylate cyclase and mitogen-activated protein kinases, while increasing intracellular Ca^2+^ levels via phospholipase C [12].

CB2 receptor effects, whether standalone or influenced by co-activation with CB1, are protective, anti-inflammatory, regenerative, and antioxidant, presenting potential benefits in oncological therapy [12].

Given the complexity of the ECS and its involvement in various physiological processes, ongoing research, particularly on compounds like CBD, holds promise for unlocking novel therapeutic strategies across multiple medical fields. The intricate interactions and roles within the ECS make the pharmacological modulation of phytocannabinoids a compelling avenue for therapeutic exploration [11,12].

**Figure 2:**
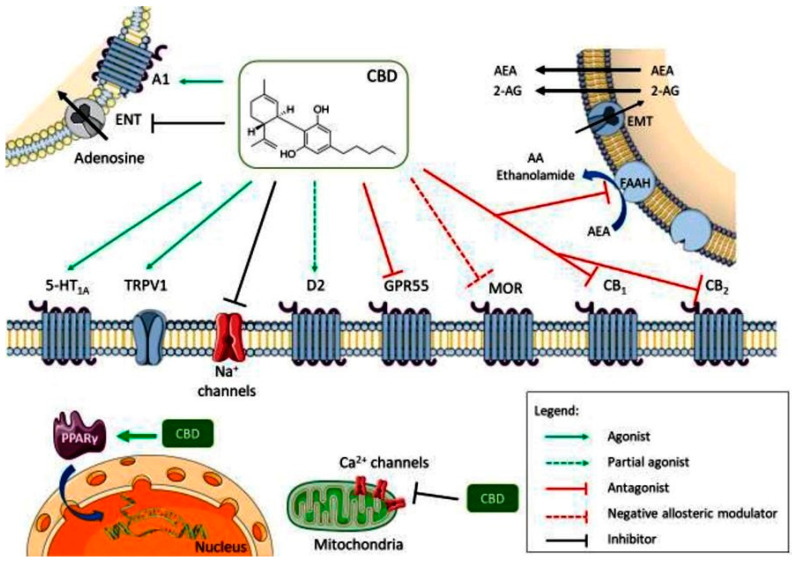
Multiple molecular targets for CBD (A1—adenosine receptor 1; ENT—equilibrative nucleotide transporter; AEA—anandamide; 2-AG—2-arachidonoylethanolamide; EMT—endocannabinoid membrane transporter; 5-HT1A—5-hidroxytriptamine 1A receptor; TRPV1—transient receptor potential vanilloid 1; D2—dopamine receptor 2; GPR55—G protein coupled receptor 55; MOR—µ opioid receptor; PPARγ—peroxisome proliferator-activated receptor gamma; CB1—cannabinoid receptor 1; CB2—cannabinoid receptor 2). Reprinted from de Almeida et al. [13] with permission from Wiley.

CBD acts as an antagonist for cannabinoid CB1 and CB2 receptors, while also inhibiting endocannabinoid degradation through the fatty acid amide hydrolase (FAAH) enzyme, leading to increased endocannabinoid levels and subsequent receptor activation. Its full agonism at 5-HT1A serotonin receptors and transient receptor potential vanilloid 1 (TRPV1) channel contributes to its anxiolytic and analgesic effects (Figure 2). Additionally, its partial agonism at D2 dopamine receptors may influence emotional memory processing in the ventral hippocampus. CBD’s full agonism at adenosine A1 receptors potentially benefits cardiac arrhythmias and myocardial ischemia/reperfusion injuries. Its negative allosteric modulation of µ opioid receptor is crucial for controlling opioid drug abuse and relapse. CBD’s agonism of intracellular peroxisome proliferator-activated receptor gamma (PPARγ) receptors leads to changes in gene transcription, positively affecting glucose and fatty acid metabolism in both animals and humans (Figure 2). Furthermore, CBD exerts an overall inhibitory effect on sodium and calcium channels, suggesting its potential as a therapeutic mechanism for epilepsy by modulating membrane electrical potential.

Currently, there are two phytocannabinoid pharmaceutical preparations approved by the US Food and Drug Administration (FDA): Epidiolex^®^—oral solution (contains only CBD) and Sativex^®^—oromucosal spray (contains both CBD and tetrahydrocannabinol (THC)). Epidiolex^®^ is an anticonvulsant preparation used to treat seizures in Lennox–Gastaut or Dravet syndrome in children aged 2 years and older. Sativex^®^ is an oromucosal spray that contains a combination of 2.7 mg THC with 2.5 mg CBD, approved for the relief of neuropathic pain, spasticity, and other associated manifestations [14,15].

There is continuous research on phytocannabinoids and the *Cannabis* plant, and knowledge about its possible medical uses is expanding. Notwithstanding, the terrain of *Cannabis* research and its application in medicine and other fields remains shaped by obstacles pertaining to law and regulation, in addition to the necessity for additional scientific investigation.

## 2. Pharmacological Profiles of Key Phytocannabinoids

### 2.1. Δ9-Tetrahydrocannabinol (THC)

THC is the primary psychoactive compound found in the *Cannabis* plant. It has a complex, bicyclic structure with a hexahydro cannabinol core, containing a cyclohexene ring (six-membered ring with alternating single and double bonds) and an oxirane ring (three-membered ring containing one oxygen atom). THC exists in different stereoisomeric forms due to the presence of chiral centers in its structure; the naturally occurring form is usually (−)-trans-Δ^9^-THC [6,7,16].

THC is well absorbed through inhalation (smoking or vaporization) and ingestion; it is highly lipophilic, as it tends to accumulate in fatty tissues; it undergoes extensive hepatic metabolism, primarily by the enzyme cytochrome P450 CYP2C9, to form the active metabolite 11-hydroxy-THC; and its metabolites are excreted in urine, with an elimination half-life ranging from several days to weeks [16,17].

THC is responsible for the distinct “high” associated with cannabis usage because it alters perception, mood, and cognition [7,16].

THC has analgesic effects and is utilized in some medical settings to treat chronic pain. It also possesses antiemetic effects, which make it useful for treating chemotherapy-induced nausea and vomiting [18,19].

Some authorities employ THC-containing medicines to treat a variety of medical ailments, such as chronic pain, nausea, and muscle stiffness. It is classified as a controlled substance in many countries due to its psychoactive properties; nonetheless, there is continuing debate and changing legislation regarding its legal status in various regions. Dronabinol and Nabilone are synthetic THC derivatives that have been approved for medical use [20].

### 2.2. Cannabidiol (CBD)

CBD (2-[(1*R*,6*R*)-3-methyl-6-prop-1-en-2-ylcyclohex-2-en-1-yl]-5-pentylbenzene-1,3-diol) is the primary non-psychoactive compound found in the *Cannabis* plant. Unlike THC, it has a tricyclic structure, and it consists of two connected rings, a phenol ring (six-membered ring with an attached hydroxyl group), and a pyran ring (six-membered ring containing oxygen). CBD also exists in different stereoisomeric forms, and the naturally occurring form is usually referred to as *trans*-CBD [21].

Unlike THC, CBD is not psychoactive. It does not produce the characteristic “high” associated with cannabis use. CBD interacts with the endocannabinoid system, but unlike THC, it does not have a high affinity for CB1 receptors, which are primarily found in the CNS and are associated with psychoactive effects. CBD’s main interactions are with CB2 receptors, which are mainly present in peripheral tissues, particularly in immune cells [6,21].

CBD has been demonstrated to have anti-inflammatory properties, which may be mediated by its interaction with the endocannabinoid system and other signaling pathways. It is said to have analgesic qualities and may be effective in pain management. It has also shown promise as an anxiolytic, with some studies indicating that it may help lower symptoms of anxiety and stress. Some research suggests that CBD may have neuroprotective properties, potentially benefiting people suffering from CNS disorders. CBD has antioxidant effects, which may contribute to its neuroprotective effects and overall health benefits [22].

CBD has gained attention for its potential to treat certain forms of epilepsy. Epidiolex^®^, a pharmaceutical-grade CBD product, has been approved for the treatment of specific types of seizures [13].

Preclinical studies have explored CBD’s potential as an anti-cancer agent, but more research is needed to fully understand its efficacy in humans [22].

### 2.3. Cannabigerol (CBG)

CBG (2-[(2E)-3,7-dimethylocta-2,6-dienyl]-5-pentylbenzene-1,3-diol) is a cannabinoid with a complex chemical structure; it contains a monocyclic core, with a phenol ring and a dihydropyran ring [6].

CBG interacts with the ECS and has a lower affinity for both CB1 and CB2 receptors compared to THC and CBD. CBG has been reported to function as a partial agonist or antagonist at CB1 receptors; this suggests that it may modulate the effects of other cannabinoids by influencing CB1 receptor activity [23].

Some studies indicate that CBG may have neuroprotective characteristics, presumably due to its interaction with the ECS and anti-inflammatory actions. CBG has been observed to stimulate appetite, like THC, which is of potential interest in medical applications [24].

Some preclinical studies suggest that CBG may have anticancer properties, including inhibiting the growth of certain cancer cells. CBG may have potential benefits for gastrointestinal health, including anti-inflammatory effects that could be relevant in conditions such as inflammatory bowel disease [23,24].

### 2.4. Other Phytocannabinoids

#### 2.4.1. Cannabichromene (CBC)

CBC (2-methyl-2-(4-methylpent-3-enyl)-7-pentylchromen-5-ol) is a cannabinoid containing a bicyclic core, like other cannabinoids, with a phenol ring and a dihydropyran ring [6].

CBC interacts with ECS, but its affinity for CB1 and CB2 receptors is relatively low compared to cannabinoids like THC [25].

CBC has demonstrated anti-inflammatory effects, which may be attributed to its interaction with the ECS and other anti-inflammatory pathways; it appears to inhibit the activation of certain inflammatory pathways and cytokines [26].

CBC has been studied for its analgesic properties and may contribute to the overall pain-relieving effects of cannabis. Some research suggests that CBC may have neuroprotective properties, potentially offering benefits in conditions affecting the CNS [26].

#### 2.4.2. Cannabinol (CBN)

CBN (6,6,9-trimethyl-3-pentylbenzo[c]chromen-1-ol) is a cannabinoid with a chemical structure that results from the degradation of THC over time or through exposure to light and air [6].

CBN interacts with the ECS but its interaction with the ECS is believed to be less potent than that of other cannabinoids. CBN is reported to have mild psychoactive effects, but they are in general much weaker than those of THC; its psychoactive effects are often considered a result of its partial agonism at CB1 receptors [27].

CBN has been suggested to have sedative properties and may contribute to the sleep-inducing effects of certain cannabis strains; some studies indicate that CBN, when combined with THC, may enhance sedation. CBN has shown some analgesic properties, and it may contribute to the overall pain-relieving effects of cannabis [27].

#### 2.4.3. Tetrahydrocannabivarin (THCV)

THCV (6,6,9-trimethyl-3-pentyl-6a,7,8,10a-tetrahydro-6H-benzo[c]chromen-1-ol) is a cannabinoid with a structure that is like THC, but with variations in its side chain and overall molecular arrangement; it contains a bicyclic core with a phenol ring and a dihydropyran ring, typical of cannabinoids [28].

THCV interacts with the ECS, and it has a higher affinity for CB2 receptors compared to CB1 receptors. THCV’s effects can vary depending on the dose and the presence of other cannabinoids. In lower doses, it may act as an antagonist to CB1 receptors, and in higher doses, it may act as a partial agonist [28].

Pharmacological profiles of the main phytocannabinoids mentioned above are summarized concisely in Table 1.

## 3. Therapeutic Applications

### 3.1. Anti-Inflammatory Effects

Cannabinoids, including CBD and THC, have been shown to possess anti-inflammatory effects. These effects are mediated through interactions with the endocannabinoid system, which plays a crucial role in regulating immune responses and inflammation throughout the body [29].

Croxford and Yamamura’s review article discussed the potential use of cannabinoids in the treatment of inflammatory diseases by modulating the immune system. It explores the anti-inflammatory properties of cannabinoids and their therapeutic implications for various inflammatory conditions, including autoimmune diseases and neuroinflammation [30].

In particular, the anti-inflammatory effects of CBD have been thoroughly researched. It interacts with immune cells, primarily situated cannabinoid receptors, such as CB2 receptors. CBD might potentially decrease the production of pro-inflammatory chemicals like cytokines and chemokines by regulating the activity of these receptors, which in turn can reduce inflammatory reactions [31].

Solinas et al. investigated the anti-inflammatory effects of CBD in glioma cells. The researchers found that CBD inhibited cell proliferation and invasion by exerting anti-inflammatory effects through multiple molecular targets, suggesting its potential as a therapeutic agent for glioma treatment [32].

Khodadadi et al. investigated the effects of CBD on cytokine storm, a severe inflammatory response observed in acute respiratory distress syndrome induced by simulated viral infection. The researchers found that CBD attenuated cytokine production and inflammation, suggesting its potential as a therapeutic agent for acute respiratory distress syndrome (ARDS) and other inflammatory lung diseases [33].

Though its mechanism of action can be different from CBD’s, THC likewise has anti-inflammatory properties. Along with other non-cannabinoid receptors implicated in inflammation, THC interacts with CB1 and CB2 receptors. THC can lessen inflammatory processes by inhibiting immune cell activation and cytokine production through these interactions [34,35].

Preclinical and clinical investigations indicate that CBD, either alone or in combination with other compounds, possesses potential antinociceptive properties across various pain conditions. The efficacy of CBD in relieving pain appears to be influenced by dosage and administration methods. Studies on healthy rodents subjected to painful stimuli, such as tail or paw pressure tests, have shown that CBD administration can mitigate nociceptive responses. Furthermore, CBD’s interaction with THC seems to vary depending on experimental conditions, with the potential for both potentiation and antagonism [36].

The antinociceptive effects of CBD appear to involve CB1, adenosine A1, and transient receptor potential ankyrin 1 receptor, but not transient receptor potential vanilloid 1 receptor. However, the findings regarding the comparative effects of CBD and THC on pain relief have been inconsistent [37]. While some studies suggest that THC may be more effective than CBD in alleviating inflammatory pain, others indicate synergistic effects when CBD is combined with morphine [38].

Safety assessments suggest that oral administration of CBD at doses ranging from 400 to 800 mg, in conjunction with fentanyl, is well-tolerated. CBD oils have shown promise in managing chronic pain, particularly in neuropathic pain models in rodents. Additionally, CBD’s modulation of dopamine and serotonin levels, possibly by reducing amygdala activation during negative emotional processes, highlights its potential in chronic pain management [39].

Studies on CBD-based oil treatment in dogs with osteoarthritis demonstrate that CBD can enhance comfort and activity levels when administered at a dose of 2 mg/kg body weight twice daily. THC also exhibits anti-inflammatory effects, which may be attributed to its inhibition of prostaglandin E2 synthesis, reduction in platelet aggregation, and stimulation of lipoxygenase [40].

Katz-Talmor et al. published a review that provides an overview of the current evidence on the use of cannabinoids for the treatment of rheumatic diseases. It discusses the anti-inflammatory properties of cannabinoids and their potential therapeutic applications in rheumatoid arthritis, osteoarthritis, and other rheumatic conditions, highlighting the need for further research in this area [39].

Overall, CBD, alone or in combination with other compounds, shows promise as a treatment for pain relief, including in chronic pain conditions. Off-label use of CBD for pain management is a possibility worth exploring further. Further research is needed to fully understand the mechanisms underlying its anti-inflammatory effects and to optimize its therapeutic use in clinical settings [30,31,37].

### 3.2. Antioxidant Effects

CBD interrupts free radical chain reactions by scavenging free radicals or converting them into less harmful forms; this mechanism helps prevent cellular damage caused by oxidative stress. This mechanism, common among antioxidants, helps mitigate oxidative stress. The phenolic structure of CBD, particularly the hydroxyl groups on the phenol ring, plays a significant role in its antioxidant properties [22]. Additionally, CBD supports the function of antioxidant enzymes by maintaining optimal levels of essential microelements like zinc and selenium, which are vital for the activity of enzymes such as superoxide dismutase and glutathione peroxidase.

In experiments on rats subjected to chronic UVA or UVB irradiation, CBD applied topically to the skin demonstrated effects on liver phospholipid metabolism, further highlighting its antioxidant properties [41].

CBD’s therapeutic potential extends to conditions associated with oxidative stress and inflammation, including cardiovascular, neurodegenerative, and metabolic diseases. At higher concentrations, CBD can activate the PPAR-γ receptors and vanilloid receptors (TRPV1 and TRPV2), contributing to its antioxidant effects. Research studies have underscored CBD’s efficacy as a potent antioxidant, suggesting its promising role in various pathological conditions characterized by oxidative stress [42].

Scotter et al. outlined the potential of targeting the endocannabinoid system for the treatment of neurodegenerative diseases, such as Alzheimer’s disease, Parkinson’s disease, and Huntington’s disease. It explores the antioxidant and neuroprotective properties of cannabinoids, including THC and CBD, and their potential as therapeutic agents for neurodegeneration [43].

Jackson et al. also discussed the neuroprotective effects of cannabinoids in CNS inflammatory diseases, such as multiple sclerosis, Alzheimer’s disease, and stroke. It explores the antioxidant and anti-inflammatory properties of cannabinoids and their potential as therapeutic agents for CNS inflammation and neurodegeneration [44].

### 3.3. Pain Management

Cannabinoids exert their analgesic effects through various mechanisms, including modulation of neurotransmitter release, inhibition of inflammatory mediators, and modulation of ion channels involved in pain signaling. Additionally, cannabinoids have been shown to have anti-inflammatory effects, which can further contribute to pain relief, especially in conditions where inflammation is a contributing factor to pain [31,45].

Research has demonstrated the potential efficacy of cannabinoids in managing chronic pain conditions such as neuropathic pain, fibromyalgia, arthritis, and cancer-related pain. They may be used alone or in combination with other analgesic medications, offering a potential alternative for patients who do not respond well to conventional treatments or who experience intolerable side effects [45,46].

Studies have revealed that non-steroidal anti-inflammatory drugs (NSAIDs), especially ibuprofen, not only affect prostaglandin synthesis but also inhibit endocannabinoid metabolism pathways, namely 2-arachidonoylglycerol and anandamide, via cyclooxygenase-2 (COX-2) and fatty acid amide hydrolase (FAAH). The inhibition of FAAH leads to increased activity of the ECS, potentially affecting appetite regulation [47].

Research indicates that NSAIDs inhibit FAAH activity, leading to enhanced ECS activity, which may contribute to the analgesic and anti-inflammatory effects of ibuprofen. Additionally, NSAIDs have been found to inhibit the cyclooxygenase of endocannabinoids by COX-2, further influencing ECS activity [47,48].

Studies have shown that NSAIDs like ibuprofen exhibit synergistic effects with endocannabinoids in pain relief, suggesting a promising avenue for analgesic therapy. Furthermore, the modulation of endorphins and endocannabinoid receptors by ibuprofen may help preserve appetite during illness, particularly in children [49].

Russo’s review article provides an overview of the role of cannabinoids in managing difficult-to-treat pain conditions. It discusses the evidence from clinical trials and observational studies regarding the efficacy and safety of cannabinoids, including THC and CBD, in various pain syndromes, such as neuropathic pain, fibromyalgia, and cancer-related pain [50].

Pergolizzi et al. review article discusses the potential of cannabinoids as analgesic agents in pain management. It explores the mechanisms underlying the analgesic effects of cannabinoids, including their interactions with the endocannabinoid system and other pain-related pathways, as well as the challenges and limitations associated with their use in clinical practice [51].

Vučković et al. provide an update on the research regarding the use of cannabinoids in pain management. It discusses the pharmacology of cannabinoids, their mechanisms of action in modulating pain perception, and the potential therapeutic applications of cannabinoids in various pain conditions, including chronic pain, neuropathic pain, and inflammatory pain [52].

Johal et al. published a systematic review and meta-analysis examining the efficacy of cannabinoids in chronic non-cancer pain management. It synthesizes the findings from randomized controlled trials to evaluate the effectiveness of cannabinoids, including THC and CBD, compared to placebo or other analgesic agents in reducing pain intensity and improving quality of life in patients with chronic pain conditions [53].

### 3.4. Neurological Disorders

Cannabinoids have gathered significant attention for their potential therapeutic applications in various neurological disorders.

CBD has shown promise in the treatment of epilepsy, particularly in rare and treatment-resistant forms such as Dravet syndrome and Lennox–Gastaut syndrome; Epidiolex^®^, a CBD-based medication, has been approved by regulatory agencies (FDA, EMA) for the treatment of the conditions mentioned above [54].

Cannabinoids have demonstrated benefits in managing symptoms associated with multiple sclerosis, including spasticity, pain, and bladder dysfunction; Sativex^®^, an oromucosal spray containing THC and CBD in a 1:1 ratio, has been approved in some countries for the treatment of multiple sclerosis-related spasticity [55].

Preclinical studies suggest that cannabinoids may have neuroprotective and anti-inflammatory effects in Parkinson’s disease; some clinical evidence and small-scale clinical studies have reported improvements in motor symptoms, sleep disturbances, and quality of life with cannabinoid-based therapies [56].

Cannabinoids, particularly THC and CBD, have demonstrated efficacy in managing neuropathic pain, which is often resistant to conventional analgesics. They modulate pain perception through their interactions with cannabinoid receptors and other neurotransmitter systems involved in pain processing [57].

While research is still in its preliminary stages, cannabinoids have shown potential in modulating neuroinflammation, oxidative stress, and neurodegeneration associated with Alzheimer’s disease and dementia [58].

Cannabinoids have also been investigated in conditions such as Huntington’s disease, amyotrophic lateral sclerosis, post-traumatic stress disorder (PTSD), and migraine, among others. While preliminary findings are promising, more research is needed to fully understand their therapeutic potential and optimize treatment protocols [59].

### 3.5. Psychiatric Conditions

Some studies suggest that cannabinoids, particularly CBD, may have anxiolytic effects. CBD has shown promise in reducing symptoms of generalized anxiety disorder, social anxiety disorder, and PTSD. However, the evidence is mixed, and more research is needed to clarify the role of cannabinoids in anxiety treatment [60].

Preclinical and clinical studies have explored the antidepressant potential of cannabinoids, primarily CBD and THC. While some findings suggest that cannabinoids may have mood-stabilizing effects and enhance serotonin signaling, the evidence is inconclusive, and further research is warranted [61].

Cannabinoids have been investigated for their potential in treating substance use disorders, including alcohol use disorder and opioid use disorder. While some studies suggest that CBD may help reduce cravings and withdrawal symptoms, the evidence is preliminary, and more research is needed to establish its efficacy and safety as a treatment for substance use disorders [62].

Cannabinoids, particularly THC, have been reported to affect sleep patterns, with some individuals experiencing improved sleep quality and duration, while others may experience disruptions, such as decreased rapid eye movement (REM) sleep. CBD has also been studied for its potential in managing insomnia and other sleep disorders, although the evidence is limited and conflicting [62].

### 3.6. Cancer Treatment Support

In recent years, research has increasingly focused on evaluating the potential of cannabinoids as antineoplastic agents. Studies indicate that cannabinoids can exert antitumor effects directly by inhibiting cell proliferation and inducing apoptosis, or indirectly by inhibiting angiogenesis, invasion, and metastasis. In vivo and in vitro research has demonstrated the efficacy of cannabinoids in modulating tumor growth, although the antitumor effects can vary depending on the type of cancer and the concentration of the drug [63].

Sarfaraz et al. provided an overview of the progress and promise of using cannabinoids in cancer treatment. It discusses the mechanisms of action by which cannabinoids exert anti-cancer effects, the preclinical and clinical evidence supporting their efficacy, and the potential challenges and opportunities for integrating cannabinoids into conventional cancer therapy [63].

Studies have linked cannabinoids to the inhibition of cancer cell growth in several cancers, often through cell-specific mechanisms. Cannabinoids intervene in tumor cell development cycles, inhibit growth, induce apoptosis, and reduce tumor cell migration and angiogenic activity. The mechanisms for these activities are not fully understood, as cannabinoid receptors interfere with various intracellular signaling pathways. The levels of endocannabinoids vary depending on the cell type and malignancy, with alterations observed in malignant tissues compared to normal tissues [64].

Historically, research dating back to the 1970s has demonstrated the antineoplastic activity of cannabinoids in various cancer models. Synthetic cannabinoids and phytocannabinoids interact with components of the endocannabinoid system, affecting the development of oncological conditions. While cannabinoids have been primarily used in palliative care for cancer patients, in vitro and in vivo models indicate their potential to modulate tumor growth, with effects contingent on the concentration of cannabinoids administered and the specific type of cancer [65].

Bifulco et al.’s review article examines the advantages/disadvantages of using cannabinoids as an antitumor strategy in cancer treatment. It discusses the molecular mechanisms underlying the anticancer effects of cannabinoids, their potential as adjuvants to standard cancer therapies, and the challenges associated with cannabinoid-based cancer treatment, such as dose optimization and patient selection [66].

For cancer patients, it is crucial to comprehend how cannabinoids control immune system interactions and other biological processes related to carcinogenesis, such as cell cycle progression, proliferation, and cell death. Additional research is necessary for this area.

### 3.7. Other Emerging Areas

The phenolic nature of cannabinoids suggests inherent antibacterial activity, but their potency against multidrug-resistant bacterial strains is particularly noteworthy. Data on the antibacterial activity of CBC, CBG, CBD, and THC have been collected. Recent studies have also investigated the potential of major cannabinoids (CBD, CBC, CBG, THC, CBN) in addressing infections caused by methicillin-resistant *Staphylococcus aureus* (MRSA), a Gram-positive bacterium responsible for a significant portion of sepsis cases and associated deaths [67].

CBG stands out as it can be produced by certain hemp chemotypes without residual THC, making it a potentially excellent and safe antiseptic agent. A recent study by Aqawi et al. in 2021 highlighted the antibacterial effects of CBG against the cariogenic bacteria *Streptococcus mutans*, demonstrating a bacteriostatic effect [68].

CBD has been extensively studied for its potential in treating diabetes and its complications. Through the activation of CB2 receptors, CBD has shown the ability to induce vasodilation in rats with type 2 diabetes mellitus. Additionally, its activation of 5-HT1A receptors has demonstrated therapeutic effects in diabetic neuropathy and accelerated wound healing in diabetic rat models, thereby protecting vascular endothelial growth factor. CBD has also been found to prevent the formation of oxidative stress in neurons of animals with diabetic retinopathy, counteracting tyrosine nitration, which can lead to glutamate buildup and neuronal cell death. Current research suggests that CBD holds promise in treating diabetes, particularly diabetic cardiomyopathy [69].

Both natural and synthetic cannabinoids have shown potential in reducing pain sensation, inflammation, inflammatory pain, and hyperalgesia and preventing secondary tissue damage in traumatic wounds. At the site of injury, cannabinoids may decrease the release of tissue activators and sensitizers, modulating nerve cells to control tissue destruction and immune cells to prevent the release of proinflammatory substances. This modulation helps minimize pain and temper post-injury responses associated with inflammatory injury.

Table 2 presents cannabinoids’ potential therapeutic applications.

## 4. Challenges and Controversies

### 4.1. Legal and Regulatory Issues

The legal and regulatory issues surrounding phytocannabinoids, particularly compounds like CBD and THC, are complex and vary widely across different jurisdictions. These issues encompass a range of concerns including legality, cultivation, distribution, sale, and use [5,20].

Due to its composition and the psychoactive effects of THC, many countries have imposed strict regulations on its use, making it challenging to access for therapeutic purposes. This has led to prohibitions and stringent legislation, hindering patients’ ability to utilize the plant for medical treatment [7,15]. Countries that have legalized cannabis often establish regulatory frameworks to oversee its production, distribution, and sale. These frameworks may include licensing requirements for cultivators, processors, and retailers, as well as regulations governing product testing, labeling, and packaging [70].

Also, the legal status of CBD, THC, hemp, hemp oil, and *Cannabis* varies throughout the EU and the USA. For example, hemp is legal throughout the EU and USA but some differences regarding the THC limit (concentration) are outlined, with a total of less than 0.3% of THC in Austria, Czech Republic, the USA, Romania, and whilst in Germany, Greece, Hungary, and Spain, less than 0.2% is permitted [20,71]. In the USA in 2018, the Farm Bill was approved and, as a result, hemp is no longer included in Schedule I of prohibited substances, but THC remains in Schedule I alongside CBD. Even so, the new Epidiolex^®^ (approved in June 2018 by the FDA) is classified in Schedule V—pharmaceutical formulations with a limited number of certain narcotics (10% CBD oily solution) [72]. Following its introduction into therapy, Epidiolex^®^ gained approval in the EU for treating Lennox–Gastaut and Dravet syndromes when used alongside clobazam. However, in certain European countries (Romania, Greece, Russia), rigid laws prevent the prescription of Epidiolex^®^ and Sativex^®^ due to a lack of marketing authorization [73]. Despite CBD’s minimal CNS side effects, its prescription remains overly restrictive. Therefore, significant legislative modifications are necessary to establish appropriate prescription protocols and potentially reclassify the active pharmaceutical ingredient to a less restrictive schedule in some countries [74].

It is crucial to note the wide array of CBD products available online and onsite, ranging from CBD oils in various concentrations (5–30%) to diverse cosmetic and daily care items like toothpaste and pain relief gel. Due to CBD’s lipophilicity, it can be blended in different proportions with oily solvents, but it is essential to ensure that concentrations align with the original product (e.g., 10%) to avoid potential side effects or exacerbated issues such as elevated transaminase levels [75,76]. Despite the therapeutic considerations, these products are often registered solely as cosmetics or even dietary supplements rather than as prescription drugs. Given the risks of adulteration and the absence of active pharmaceutical ingredient (API) regulation in online CBD products, it is imperative to have prescription-based alternatives available in all countries to mitigate the use of such products. This is particularly relevant given the proliferation of online platforms selling CBD products [75,76].

As can be noticed in Table 3, there has been a notable increase in the development of various formulations containing CBD, each serving different purposes. However, this proliferation may undermine the credibility of the substance, as some formulations lack correlation with CBD’s mechanism of action, or the reported results are inconsistent. Additionally, the concentration of CBD in certain formulations may not align with therapeutic efficacy, posing a disadvantage. Nonetheless, these products have garnered success partly due to the inclusion of “established” ingredients with known therapeutic benefits, with adulteration of dietary supplements currently being a big public health issue.

The laws and regulations governing phytocannabinoids are subject to change as scientific understanding evolves, public attitudes shift, and policymakers respond to emerging issues and challenges.

### 4.2. Standardization of Phytocannabinoid Products

Standardization efforts should align with regulatory requirements set forth by governing bodies such as the FDA or the European Medicines Agency (EMA). Standardization of phytocannabinoid products is essential for ensuring consistency, quality, and safety across different formulations and brands. Standardization involves accurately measuring and labeling the content of major cannabinoids such as CBD and THC. This ensures that consumers know exactly what they are purchasing and consuming [5,77].

Terpenes are aromatic compounds found in *Cannabis* that contribute to its flavor and aroma, as well as potential therapeutic effects. Standardizing the terpene profile helps maintain consistency in sensory attributes and may also impact the “entourage” effect—the synergistic interaction between cannabinoids and terpenes [9,78].

Phytocannabinoid products should undergo rigorous testing for contaminants such as pesticides, heavy metals, microbial organisms, and residual solvents. Standardizing limits for these contaminants ensures product safety and compliance with regulatory requirements [79].

### 4.3. Potential for Abuse and Dependence

THC is the primary psychoactive compound found in cannabis and is responsible for the “high” or euphoric effects commonly associated with marijuana use. These psychoactive effects can contribute to its potential for abuse, as individuals may seek out the pleasurable sensations induced by THC consumption [17].

Chronic use of THC-containing cannabis products can lead to the development of dependence and addiction in some individuals. While the overall risk of addiction to THC is lower compared to substances like opioids, amphetamines, or alcohol, it is still a concern, particularly for individuals who use cannabis frequently or in high doses [16,80].

Unlike THC, CBD does not produce psychoactive effects and is not associated with the same potential for abuse or dependence. Research suggests that CBD may even have potential therapeutic effects in reducing addiction to other substances, such as opioids, alcohol, or nicotine [81].

Some cannabis products contain a combination of THC and CBD, which can affect their abuse potential. The presence of CBD may mitigate some of the psychoactive effects of THC and reduce its potential for abuse, but this varies depending on the ratio of THC to CBD and other factors. CBD is generally considered to have a low potential for abuse and dependence, and THC-containing cannabis products pose a greater risk, particularly when used chronically or in high doses [82].

The potential for abuse and dependence associated with phytocannabinoids can vary widely among individuals. Factors such as genetic predisposition, underlying mental health conditions, environmental influences, and patterns of use all play a role in determining an individual’s risk of developing problematic cannabis use [5,7].

## 5. Future Directions

### 5.1. Ongoing Research and Preclinical Trials

Ongoing research and preclinical trials hold high promise for unlocking the therapeutic potential of phytocannabinoids and expanding treatment options for various medical conditions. As research progresses and regulatory hurdles are addressed, we can expect to see a wider range of cannabinoid-based therapies becoming available in the future.

Recent advancements include the growing recognition of the therapeutic potential of minor cannabinoids beyond CBD and THC, such as CBG, CBN, and CBC. Researchers are also developing novel delivery methods, including transdermal and topical systems, to improve bioavailability and targeted delivery.

Given the limited availability of prescription pharmaceutical products authorized by regulatory bodies such as the FDA and EMA, there exists significant potential for stable CBD products to enter the pharmaceutical market. Several pharmaceutical formulations incorporating varying levels of CBD have been developed, with some undergoing formulation and preformulation studies, and others progressing through preclinical trials. Researchers are developing novel formulations to enhance the delivery and bioavailability of cannabinoids. This includes nanoemulsions, liposomes, and micelles, which improve solubility and stability, leading to more effective drug delivery [8,83].

*Preformulation and formulation studies—orodispersible formulations (ODxs)*

**Figure 3:**
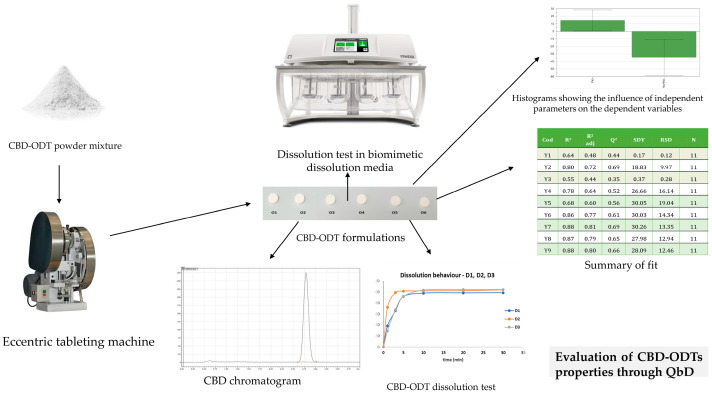
Application of QbD for the optimization of CBD-ODT formulations.

Considering the need to address pediatric patients, formulations tailored to specific age groups are essential; one such formulation intended for children is the orodispersible formulation. A 10 mg CBD orodispersible tablet (CBD-ODT) has been developed using Quality by Design (QbD) principles to optimize formulation parameters such as disintegration time, crushing strength, friability, and the release profile of the API at different intervals (Figure 3). Independent variables, including the choice of solid carriers, were carefully considered [84,85].

However, a significant challenge encountered in developing ODT with CBD is the in vitro dissolution test, owing to the API’s low solubility in water-based media. To address this issue, varying amounts of surfactants (such as sodium lauryl sulfate or Tween 20) were incorporated to establish an appropriate dissolution medium. Notably, no further in vivo evaluation results have been reported for these two CBD-ODT studies [85,86].

*Homogenous liquid pharmaceutical formulations*

The choice of pharmaceutical formulations is closely linked to the classification of CBD in the second biopharmaceutical class (BCS II), characterized by low solubility in water and high permeability. Consequently, extensive research has been conducted on oily solutions utilizing several types of vegetable oils and assessing their stability under different conditions.

Compared to solid pharmaceutical formulations, liquid formulations are generally less stable, with decomposition rates increasing over time, with temperature variations, and due to exposure to atmospheric oxygen. When developing oily solutions, careful consideration must be given to the choice of oil. For instance, if hemp oil is selected, there is a risk of cumulative CBD content, combining both the CBD intentionally added and the naturally occurring CBD present in hemp (typically less than 0.5%). Therefore, it may be prudent to consider using a neutral oil with a reduced acidity index to minimize this accumulation [87,88].

*Heterogenous pharmaceutical formulations*

In a study by Nakano et al., a comparison was made between CBD oil and CBD nanoemulsion (consisting of Tween 20, ethanol, and water). The aim was to assess plasma concentration–time curves. The results showed that the CBD nanoemulsion exhibited decreased t_max_ and t_1/2_ values, while c_max_ increased compared to the CBD oil [89].

In another study conducted by Stan et al., a microemulsion of berberine, supplemented with CBD, was developed as a targeted drug delivery system for treating irritable bowel syndrome with diarrhea. The pharmacokinetic performance was evaluated in Wistar male rats, revealing that a ketogenic diet could lead to overexpression of CB1 receptors, offering a potential target for IBS-D-specific delivery of berberine [90].

*Nanoformulations with CBD*

With a focus on targeted drug delivery, nanoformulations like liposomes present promising avenues in pharmaceuticals. In a study by Fu et al., liposomes incorporating CBD and 20(*S*)-protopanaxadiol, along with glycosyl-modified co-loading liposomes, were developed to enhance the tolerability and efficacy of CBD in rats with breast cancer [91].

In another study, Shilo Benjamini et al. administered liposomal CBD (5 mg/kg) to a dog suffering from testicular neoplasia, hip and elbow osteoarthritis, and severe cervical pain. Following the administration of liposomal CBD, the dog exhibited improved pain relief and increased collar activity scores for up to three weeks. Subsequently, a second dose of CBD liposomal injection (3 mg/kg) was given after three weeks. Unfortunately, the dog passed away the following day due to its advanced age and multiple pathologies, compounded by extreme heat. A necropsy revealed severe cervical disc protrusion and heat stroke with spinal hematoma, none of which were attributed to the liposomal CBD administration [92].

**Figure 4:**
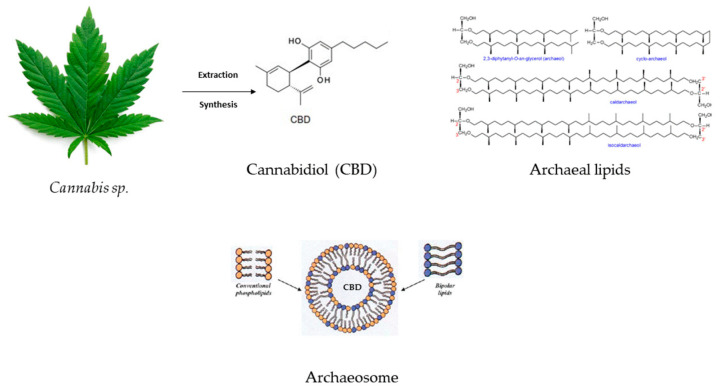
CBD’s inclusion in archaeal lipids forming a CBD archaeosome with improved bioavailability and gastrointestinal stability.

Sedlmayr et al. developed CBD liposomes and archaeosomes (Figure 4) (using archaeal lipids–diether and tetraether lipids with different headgroups and mono and di-hexoses) to improve the API storage and oral delivery because CBD’s bioavailability via oral administration is reduced. The study underscored the enhanced stability of CBD-loaded archaeosomes and increased oral stability in the simulated gastrointestinal tract. Another advantage of archaeosomes is the lower CBD dose that needs to be encapsulated in order to obtain the same therapeutic effect as in the case of CBD oils or CBD-THC sprays. The possibility of archaeosomes’ lyophilization might ensure long-term storage [93].

Volmajer Valh et al. encapsulated CBD in liposomes to evaluate their potential sanitary utility by coating different viscous surfaces with CBD liposomes outlining both antimicrobial (on Gram + bacteria) and antioxidant effects. The product was developed as a biodegradable hygiene product to overcome menstrual symptoms [94].

Another attempt at liposome development was conducted by encapsulating the hemp extract in order to highlight the use of liposomal hemp in the treatment of cachexia in rats, showing good results since two out of seven mice showed improved body weight at a low dose of 0.2 mg of liposomal hemp extract, whilst when a higher dose was administrated (1 mg) four out of seven mice survived in comparison with the control group, where only one mouse survived [95].

Franze et al. designed liposomal formulations with lidocaine and CBD in fixed combination to evaluate their potential in neuropathic pain treatment. For a better comparison, two methods were used to develop liposomes, the pH gradient method and encapsulation in micelles, with the latter showing improved skin permeation and retention in the dermis [96].

### 5.2. Exploration of Novel Phytocannabinoids

The exploration of novel phytocannabinoids is rapidly evolving, offering exciting prospects for future therapeutic applications. Beyond well-established compounds like THC and CBD, the quest for novel cannabinoids widens the scope of potential treatments. Each cannabinoid, with its unique chemical structure, interacts differently with the ECS, suggesting tailored therapeutic effects for specific conditions. This exploration seeks to harness similar benefits while circumventing associated drawbacks [5,21].

The current strategies for exploration encompass various avenues: botanical research (identifying and characterizing novel cannabinoids present in different cannabis strains), synthetic chemistry (chemical synthesis enables the creation of cannabinoid analogs with desired therapeutic properties), metabolic engineering (modifying the biosynthetic pathways of cannabis plants encourages the production of specific novel cannabinoids), selectivity to receptor subtypes (identifying cannabinoids with specific receptor selectivity holds promise for more targeted therapies with fewer side effects), non-psychoactive cannabinoids (exploring cannabinoids devoid of THC’s psychoactive effects while retaining therapeutic benefits broadens their potential applications and addresses concerns about misuse), water-soluble formulations (developing water-soluble cannabinoid formulations enhances bioavailability and facilitates administration through various routes) [8,97].

Despite its potential, legal restrictions and societal stigma surrounding cannabis hinder investment in research and development. Complex regulatory frameworks further complicate exploration efforts. Rigorous preclinical and clinical trials are imperative to establish safety and efficacy before therapeutic implementation.

### 5.3. Integration into Mainstream Medicine

Due to stringent legislation, only a limited number of pharmaceutical formulations and medicines containing cannabinoids from the *Cannabis* plant have been marketed. These cannabinoids are often categorized alongside the plant itself or THC, the compound responsible for the addictive effects, particularly when found in higher concentrations in parts such as leaves and flowers. Illicit demands typically involve *Cannabis* plants or resins with high THC content, like hashish. Consequently, cannabinoids remain prohibited in many countries, despite studies indicating THC’s role in addiction.

Integrating cannabis-derived medicines has been challenging, with the first formulation approved in 2010—Sativex^®^. It was not until eight years later that Epidiolex^®^ was marketed in the USA and later in the EU. The limited availability of *Cannabis* products in the pharmaceutical market can also be attributed to their high lipophilicity, making them suitable for formulations like oily solutions, soft capsules, emulsions, and liposomes [5,8].

CBD and THC should be accessible to patients with special prescriptions due to their potential benefits, especially for individuals with epilepsy refractory to other medications. Currently, two formulations, Epidiolex^®^ (CBD oily solution 10%) and Sativex^®^ spray (a CBD-THC combination), are approved for specific conditions and available via prescription in the USA and the EU [14,15].

However, not all European countries have access to these formulations due to disparate national laws. This discrepancy leads to increased reliance on online stores, which poses risks of adulteration, lack of API, impurities, and inadequate stability testing. Moreover, the absence of pharmacovigilance rules for online CBD products hinders the reporting of new side effects [70,74].

To mitigate risks, products containing THC should be strictly available via prescription, with similar regulations applied to CBD products, despite their lower addiction risk. Prescription retention in pharmacies after each refill or release can aid in monitoring usage. Additionally, THC products should adhere to state-specific rules, potentially requiring a special prescription to curb misuse [70].

### 5.4. Implications for the Future of Pharmacotherapy

Phytocannabinoids offer a wide range of therapeutic applications, including pain management, neurological disorders, inflammation, psychiatric conditions, and more. Phytocannabinoids may provide alternatives to traditional medications, particularly for conditions where current treatments have limitations or adverse effects [8].

Phytocannabinoids represent a promising frontier in pharmacotherapy, offering diverse therapeutic applications and a relatively favorable safety profile. Further research and development efforts are warranted to fully exploit their potential to enhance human health. Phytocannabinoids hold the potential to address emerging health challenges, such as antibiotic resistance and inflammatory diseases. Their antimicrobial and anti-inflammatory properties make them valuable candidates for combating resistant infections and modulating inflammatory pathways. Studies have demonstrated the antimicrobial properties of essential oils and cannabis extracts, particularly cannabinoids such as CBC and CBG. These compounds show promise as natural antimicrobial agents, capable of enhancing the effectiveness of antibiotics against resistant bacteria [98].

Recent attention has also focused on cannabinoids’ anti-inflammatory properties and their role in modulating the ECS in infectious conditions. The ECS contributes to immunopathology and inflammation modulation in respiratory viral infections by regulating apoptosis, cell proliferation, and pro-inflammatory cytokine production. Researchers have investigated the ECS’s involvement in infections with respiratory syncytial virus, HIV, hepatitis C virus, and herpes simplex virus. Studies suggest that cannabinoid receptors CB1 and CB2, particularly activated by CBD, can inhibit cytokine activation, exerting antiviral effects. CBD has shown promise in suppressing virus replication, limiting the proliferation of infected cells, and reducing neuroinflammation in viruses such as Theiler’s murine encephalomyelitis virus and Kaposi’s sarcoma-associated herpesvirus, as evidenced by previous research findings [99].

Phytocannabinoids have shown promise in combination therapies, enhancing the effectiveness of existing medications or targeting multiple pathways involved in disease pathogenesis. This approach could lead to synergistic effects and improved treatment outcomes.

## 6. Conclusions

The ECS is a complex and active messenger system present across various organs and tissues, offering promising avenues for the development of new drugs targeting conditions that are currently inadequately treated or untreated. Alongside direct or indirect agonists and antagonists of cannabinoid receptors, enzymes involved in ECS functions are also being targeted to produce beneficial effects with minimal psychotropic side effects.

Recent findings on the anti-inflammatory and analgesic properties of CB2 receptors hold potential for treating diseases linked to ECS activation. Another class of indications pertains to enhancing ECS signaling, which includes addiction (such as smoking, drug, and alcohol abuse) and metabolic diseases (like obesity, fatty liver disease, and diabetes). These disorders share a common component—appetite—which can be addressed with selective CB1 receptor antagonists.

The development of new drugs necessitates in vivo pharmacological studies to determine the structure–activity relationships of candidate agents, ensuring minimal psychotropic side effects. Furthermore, given the abundance of CB1 receptors in the brain, understanding the consequences of long-term cannabinoid receptor inhibition is crucial before introducing new agents.

The discovery of non-CB1/non-CB2 receptors in cardiovascular and other tissues, along with evidence showing that endocannabinoids act on vanilloid receptors and other receptors, underscores the ECS’s potential in managing various pathological diseases once its mechanisms are better understood.

The body’s ECS plays essential roles in numerous metabolic, immunological, inflammatory, behavioral, and cognitive processes, including appetite, satiety, and affect regulation. It also regulates cellular apoptotic processes and mitochondrial and brain functions. The complexity of utilizing cannabinoids for medical purposes has been a topic of interest for decades, especially in specialties where inflammation control is crucial for both acute and chronic conditions associated with adult mortality.

Their anti-inflammatory effects mediated through interactions with the ECS offer potential treatments for inflammatory diseases, autoimmune conditions, and neuroinflammation. Additionally, cannabinoids demonstrate antioxidant effects, which can mitigate oxidative stress and contribute to the management of cardiovascular, neurodegenerative, and metabolic disorders.

In pain management, cannabinoids have shown efficacy in alleviating chronic pain conditions, including neuropathic pain, fibromyalgia, and cancer-related pain, either alone or in combination with other analgesic medications. Moreover, cannabinoids hold potential in the treatment of neurological disorders such as epilepsy, multiple sclerosis, Parkinson’s disease, and Alzheimer’s disease, offering neuroprotective and anti-inflammatory effects.

Furthermore, cannabinoids exhibit promising prospects in managing psychiatric conditions, including anxiety disorders, depression, substance use disorders, and sleep disturbances. In the realm of cancer treatment support, cannabinoids have demonstrated antineoplastic effects by inhibiting tumor growth and metastasis, although further research is necessary to optimize treatment protocols.

The legal and regulatory landscape surrounding phytocannabinoids remains complex and varies widely across different jurisdictions. While some countries have implemented stringent regulations, others have legalized cannabis for medical or recreational use, albeit with varying restrictions. The legal status of cannabinoids, hemp, and cannabis-derived products differs significantly among regions, posing challenges for patients and healthcare providers seeking access to these treatments.

Despite these challenges, there is a growing recognition of the therapeutic potential of phytocannabinoids, particularly CBD and THC, in treating various medical conditions. However, integrating cannabis-derived medicines into mainstream medicine has been hindered by regulatory barriers and the limited availability of pharmaceutical formulations. Nevertheless, recent advancements in formulation technologies, such as orodispersible tablets, nanoemulsions, and liposomal delivery systems, hold promise for enhancing the bioavailability and therapeutic efficacy of cannabinoids.

Looking ahead, ongoing research and trials continue to explore the therapeutic potential of phytocannabinoids, including minor cannabinoids beyond CBD and THC. Standardization efforts are essential to ensure consistency, quality, and safety across different formulations and brands. Moreover, exploring novel cannabinoids, developing water-soluble formulations, and investigating targeted drug delivery systems are key areas of interest for future research and development.

Phytocannabinoids offer diverse therapeutic applications, ranging from pain management to neurological disorders and inflammatory diseases. Their antimicrobial and anti-inflammatory properties make them valuable candidates for combating antibiotic resistance and modulating inflammatory pathways. By leveraging the synergistic effects of combination therapies and targeting multiple disease pathways, phytocannabinoids hold immense potential to revolutionize the future of pharmacotherapy and improve human health outcomes. However, further research, regulatory clarity, and standardized protocols are needed to fully unlock their therapeutic benefits and ensure safe and effective use in clinical practice.

## Figures and Tables

**Table 1:** Pharmacological profiles of the main phytocannabinoids [8,12].

Phytocannabinoids	Interactions	Effects
Δ^9^-Tetrahydrocannabinol (THC)	- THC primarily interacts with CB1 receptors (predominantly found in CNS). - It binds to CB1 receptors, leading to psychoactive effects associated with cannabis use. - THC may interact with CB2 receptors, particularly in immune cells.	- psychoactive effects, including euphoria, altered perception, and mood changes. - analgesic (pain-relieving) properties. - stimulation of appetite. - potential anti-nausea effects.
Cannabidiol (CBD)	- CBD has a low affinity for CB1 receptors and does not produce the psychoactive effects associated with THC. - It interacts with CB2 receptors, mainly found in peripheral tissues, and influences other non-cannabinoid receptors, such as 5-HT1A receptors.	- anti-inflammatory properties. - analgesic and antispasmodic effects. - anxiolytic properties. - potential antiepileptic effects. - neuroprotective properties.
Cannabigerol (CBG)	- CBG has a low affinity for both CB1 and CB2 receptors. - It may act as a partial agonist or antagonist at CB1 receptors. - CBG also interacts with other receptors, such as TRPV1 and TRPA1 receptors.	- anti-inflammatory properties. - neuroprotective effects. - potential antimicrobial properties.
Cannabichromene (CBC)	- CBC interacts with both CB1 and CB2 receptors, although its affinity is relatively low. - It may modulate the activity of other receptors, such as TRPA1 and TRPV1 receptors.	- anti-inflammatory and analgesic properties. - potential neuroprotective effects. - potential antimicrobial properties.
Cannabinol (CBN)	- CBN is a degradation product of THC and has a lower affinity for CB1 receptors than THC. - It may interact with CB2 receptors to some extent.	- mild psychoactive effects. - sedative properties. - potential anti-inflammatory and analgesic effects.
Tetrahydrocannabivarin (THCV)	- THCV has variable effects on CB1 receptors, acting as an antagonist at lower doses and a partial agonist at higher doses. - It has a higher affinity for CB2 receptors.	- anticonvulsant properties. - neuroprotective effects. - potential appetite-suppressant effects.

**Table 2:** Cannabinoids’ therapeutic applications.

Cannabinoid	Therapeutic Applications
Δ^9^-Tetrahydrocannabinol (THC)	pain management, anti-inflammatory, nausea reduction, appetite stimulation, muscle spasm reduction
Cannabidiol (CBD)	anti-inflammatory, antioxidant, pain management, neurological disorders (e.g., epilepsy, Parkinson’s disease), psychiatric disorders (e.g., anxiety, depression), cancer treatment support, acne treatment, neuroprotective effects
Cannabigerol (CBG)	anti-inflammatory, pain management, neuroprotective, potential treatment for glaucoma, antimicrobial properties
Cannabichromene (CBC)	anti-inflammatory, pain management, potential antidepressant effects, neuroprotective properties
Cannabinol (CBN)	mild sedative, pain relief, potential antibacterial effects, appetite stimulant
Tetrahydrocannabivarin (THCV)	appetite suppression, potential treatment for diabetes, neuroprotective effects

Note: The therapeutic applications mentioned are based on current research findings and may not be exhaustive.

**Table 3:** List of pharmaceutical forms where CBD is incorporated in different concentrations (food/dietary supplements and cosmetical products).

Product Name	Pharmaceutical Form	CBD Concentration	References
CBD oil	oily solution	5, 10, 15, 25, 30%	https://www.legalize-it.ro/ulei-cbd-canabis/
Hand cream	semisolid formulation (cream)	0.2%	https://www.legalize-it.ro/ulei-cbd-canabis/unguent/crema-de-maini-39-cibdol-39-200mg-cbd-100ml-p25054/
Mouthwash	solution	0.1%	https://www.legalize-it.ro/search/apa%20de%20gura?q=apa+de+gura
Chewable tablets	tablets	600, 1200, 1800 mg	https://www.legalize-it.ro/search/tablete?q=tablete
Toothpaste	paste	0.133%	https://www.legalize-it.ro/ulei-cbd-canabis/unguent/pasta-39-pharmahemp-39-de-dinti-39-cosmohemps-cbd-39-100mg-cbd-75ml-p24680/
Nasal CBD spray	solution with special administration	0.8%	https://www.legalize-it.ro/ulei-cbd-canabis/unguent/spray-nasal-39-cibdol-39-50mg-cbd-10ml-p25056/
Soothing/calming gel	semisolid formulation (gel)	0.1%	https://www.legalize-it.ro/ulei-cbd-canabis/gel-39-justcbd-39-pentru-dureri-musculare-1000mg-cbd-p26872/

All examples from Table 3 were accessed on 6 March 2024.

## Abbreviations

API—active pharmaceutical ingredient; CB—cannabinoid; CBC—cannabichromene; CBD—cannabidiol; CBE—cannabielsoin; CBG—cannabigerol; CBL—cannabicyclol; CBN—cannabinol; CBT—cannabitriol; CNS—central nervous system; COX-2—cycloxygenase 2; ECS—endocannabinoid system; EMA—European Medical Agency; FAAH—fatty acid amide hydrolase; FDA—Food and Drug Administration; NSAID—non-steroidal anti-inflammatory drug; ODT—orodispersible tablet; QbD—Quality by Design; PPARγ—peroxisome proliferator-activated receptor gamma; PTSD—post-traumatic stress disorder; THC—tetrahydrocannabinol; Δ8-THC—(−)-Δ8-trans-tetrahydrocannabinol; THCV—tetrahydrocannabivarin; TRPV—transient receptor potential vanilloid.
